# Acute and Chronic Fetal Anemia as a Result of Fetomaternal Hemorrhage

**DOI:** 10.1155/2014/296463

**Published:** 2014-04-07

**Authors:** Paul Singh, Tara Swanson

**Affiliations:** ^1^Division of Maternal-Fetal Medicine, Department of Obstetrics and Gynecology, University of Missouri School of Medicine, Kansas City, MO 64108, USA; ^2^Division of Pediatric Cardiology, Department of Pediatrics, University of Missouri School of Medicine, Children's Mercy Hospital, Kansas City, MO 64108, USA

## Abstract

*Introduction.* Fetomaternal hemorrhage represents a transfer of fetal blood to the maternal circulation. Although many etiologies have been described, most causes of fetomaternal hemorrhage remain unidentified. The differentiation between acute and chronic fetomaternal hemorrhage may be accomplished antenatally and may influence perinatal management. *Case.* A 36-year-old gravida 6 para 3 presented at 37 and 5/7 completed gestational weeks with ultrasound findings suggestive of chronic fetal anemia such as right ventricular enlargement, diminished cerebral vascular resistance, and elevated middle cerebral artery end-diastolic velocity. On the other hand, signs of acute fetal decompensation such as deterioration of the fetal heart tracing, diminished biophysical score, decreased cord pH, and increased cord base deficit were noted. Following delivery, the neonate's initial hemoglobin was 4.0 g/dL and the maternal KB ratio was 0.015 indicative of a significant fetomaternal hemorrhage. *Discussion.* One should consider FMH as part of the differential diagnosis for fetal or immediate neonatal anemia. We describe a unique case of FMH that demonstrated both acute and chronic clinical features. It is our hope that this case will assist practitioners in differentiating acute FMH that may require emergent delivery from chronic FMH which may be able to be expectantly managed.

## 1. Introduction

Fetomaternal hemorrhage (FMH) refers to the passage of fetal blood into the maternal circulation before or during delivery. The incidence of FMH is between 1/300 and 1/1500 pregnancies and has been reported to account for approximately 0.04 percent of stillbirths [1]. Although a number of etiologies have been associated with FMH, most causes remain unidentified [2]. Although the differentiation between acute and chronic FMH may be clinically problematic, its distinction can significantly influence perinatal management. We describe a unique case of FMH that demonstrated both acute and chronic clinical features.

## 2. Case

**Figure 1:**
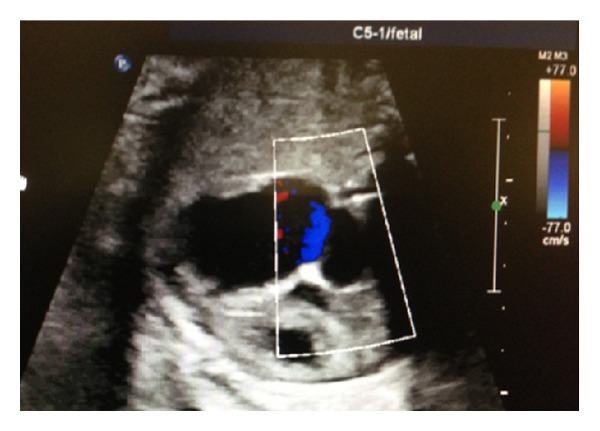
Fetal echocardiography demonstrating right atrial and ventricular enlargement.

**Figure 2:**
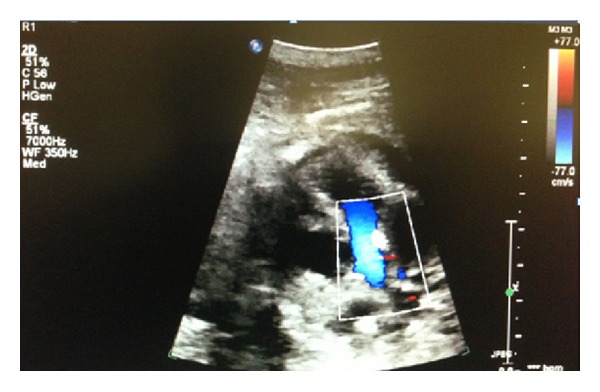
Note the antegrade Doppler flow present in the left ventricular outflow tract.

**Figure 3:**
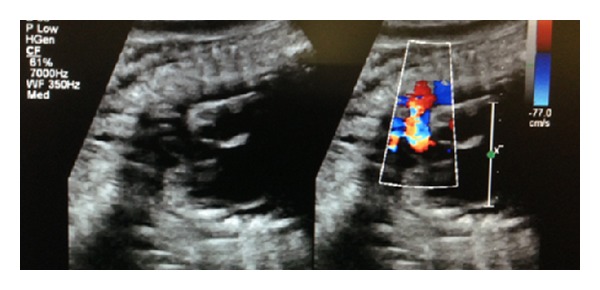
Note the torrential retrograde flow and aliasing seen in the distal aortic arch.

**Figure 4:**
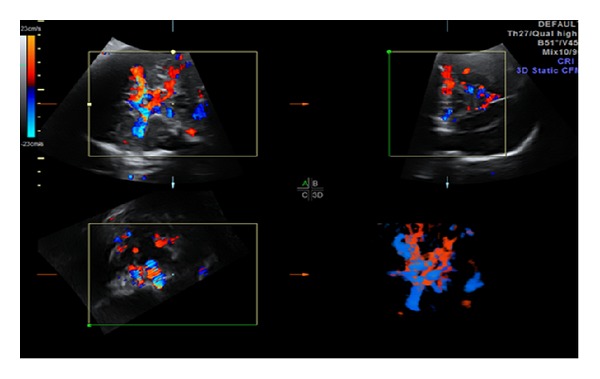
Increased brain vascularity seen on 2D and 3D ultrasound.

**Figure 5:**
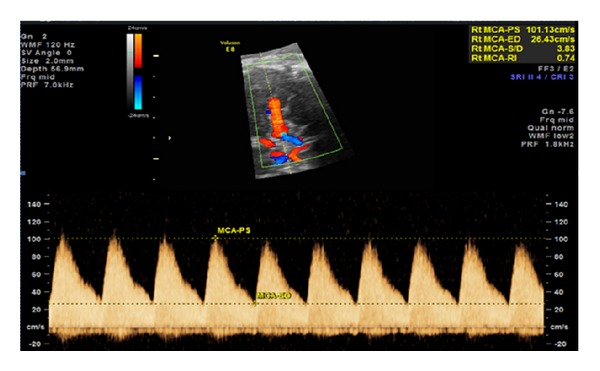
Increased middle cerebral artery end-diastolic velocities suggestive of brain sparing.

A 36-year-old gravida 6 para 3 at 37 and 5/7 completed gestational weeks was referred to our perinatal diagnostic center for fetal right atrial enlargement. Fetal echocardiography confirmed a dilated right atrium and ventricle (Figure 1). Although antegrade flow was present in the left ventricular outflow tract (Figure 2) and proximal aortic arch, this was followed by torrential retrograde flow in the distal aortic arch (Figure 3). Color Doppler interrogation of the fetal brain showed markedly increased vascularity within the fetal brain at the level of the circle of Willis (Figure 4). Elevated middle cerebral artery peak systolic velocities were also noted (Figure 5). The patient was kept overnight for observation due to a nonreactive fetal heart rate tracing. A biophysical profile was performed the following morning and revealed a score of 2/10 with recurrent fetal decelerations. Emergent cesarean delivery was undertaken. A baby girl was delivered with Apgar scores of 1, 2, and 7, with an arterial cord pH of 6.95 and a base deficit of 12.5. The infant appeared floppy at birth with no initial respiratory effort. The neonate was resuscitated, intubated, stabilized, and transferred to the neonatal intensive care unit. The neonate's initial hemoglobin, reticulocyte count, and nucleated red blood cell count were 4.0 g/dL, 6.0%, and 360 per 100 white blood cells, respectively. The maternal KB ratio was 0.015 which, corresponding to a fetomaternal hemorrhage of approximately 75 mL, assuming an estimated maternal circulating volume of 5 liters. The placenta did not show any gross or histopathologic abnormalities. After multiple blood transfusions, the neonate's hemoglobin improved and eventually stabilized. Eventually, the neonate was discharged home on day of life 21.

## 3. Discussion

Fetomaternal hemorrhage (FMH) was first described during the 1940s and 1950s [3–6]. Although often thought of as separate and distinct circulations, a baseline amount of bidirectional flow across the placenta between mother and fetus is considered physiologic. FMH, however, represents a significant loss of fetal blood cells into the maternal circulation. Although there is no universally accepted definition of the degree of fetal erythrocyte transfer that constitutes FMH, a wide range of blood volumes ranging between 10 and 150 mL have been proposed [7]. Thirty milliliters of FMH is often used as the threshold for administration of Rhogam in order to prevent Rh sensitization. Massive FMH has been described when blood volumes greater than 80 mL are transferred. Unfortunately, the degree of FMH has not been shown to directly correlate with perinatal morbidity or mortality. Indeed, Wylie and d'Alton concluded that attempting to universally define the amount of fetal blood volume transferred above which fetal injury occurs is not worthwhile [8]. Definitions of FMH relying upon a percentage of fetal blood lost to the maternal circulation have also proven problematic since normal values for fetal blood volume have not reliably been determined for various gestational ages [9, 10].

FMH is hypothesized to occur via disruption of the placental trophoblast, leading to entry of fetal erythrocytes into the maternal circulation. A number of obstetrical events have been associated with FMH including external cephalic version, amniocentesis, abdominal trauma, placental abruption, placental tumors, and manual removal of the placenta [11–21]. Often, however, FMH occurs without a precipitating factor. In a review of 134 cases with FMH greater than 50 mL, Giacoia identified 111 cases that did not have an antecedent triggering event [2]. Rather, decrease or absence of fetal movement was the most common antenatal presentation of FMH [2]. Other clinical findings associated with FMH included neonatal anemia, stillbirth, hydrops fetalis, nonreassuring fetal heart rate tracing, intrauterine growth restriction, and fetal tachyarrythmias. Interestingly, despite its frequent relationship with fetal anemia, Giacoia observed that a sinusoidal fetal heart rate pattern occurred in only approximately 10% of FMH cases [2].

Several diagnostic modalities for FMH have been described. First described by Kleihauer et al. in 1957, the most frequently used is the acid elution test [22]. Often referred to as the Kleihauer-Betke (KB) stain, this test is based on the principle that fetal hemoglobin is resistant to acid elution compared to adult hemoglobins. Following incubation in an acid based solution, a small sample of maternal venous blood is stained with erythrosine B. Erythrocytes containing fetal hemoglobin appear red while adult red blood cells are colorless. The number of fetal cells is then counted and reported as a percentage of the amount of adult cells. Unfortunately, the time required for availability of results varies considerably depending upon the technician's experience or laboratory capabilities [23, 24]. Furthermore, underestimation may occur as a result of diminished fetal hemoglobin that sometimes occurs in fetal red blood cells. Overestimation of FMH may also occur if a disproportionate number of adult red blood cells contain fetal hemoglobin, as is seen in cases of hereditary persistence of fetal hemoglobin, sickle-cell anemia, and beta thalassemia trait. Flow cytometry is an alternative diagnostic method for FMH that determines the number of fetal erythrocytes in a maternal venous blood sample by the measurement of monoclonal antibodies binding to fetal hemoglobin [25, 26]. The advantages of flow cytometry over the KB stain for the diagnosis of FMH include improved interpreter objectivity, faster turnaround times, and better accuracy. Unfortunately, very few hospitals currently utilize flow cytometry technology due to increased cost and decreased availability of laboratory technicians familiar with this technique.

The most frequently used formula to determine the volume of fetal erythrocytes lost to the maternal circulation is simply to multiply the percentage of fetal blood cells by the estimated total maternal circulating volume. Most formulas use an average maternal blood volume of 5.0 liters. Unfortunately, maternal blood volumes can vary significantly based on maternal weight, thus resulting in considerable variation in the determination of fetal blood volume lost [27]. Furthermore, the degree of FMH may be underestimated in cases of maternal-fetal ABO incompatibility and isoimmunization since the fetal blood cells that enter the maternal circulation are rapidly cleared by the maternal reticuloendothelial system.

Unfortunately, the determination of the timing of FMH is difficult to ascertain. Neither the KB stain nor flow cytometry addresses the question of when the fetal cells entered the maternal circulation. Instead, clinical and laboratory parameters are utilized to assess the chronicity of FMH. In acute FMH, rapid fetal blood loss is associated with perinatal hypoxia or acidemia that may clinically manifest as fetal or immediate neonatal anemia, nonreassuring fetal heart rate patterns, hemodynamic neonatal instability, and even stillbirth or neonatal death. In cases of chronic FMH, the fetus reacts by compensatory mechanisms of enhanced hemopoetic activity with resultant increased production of erythrocyte precursors such as erythroblasts, reticulocytes, and nucleated red blood cells [2]. In addition, vascular redistribution of fetal blood flow may occur with increased shunting of blood away from the somatic circulation to the brain, heart, and adrenal glands. The vascular alterations that occur in response to chronic fetal anemia are often referred to as the brain-sparing effect and may be documented sonographically as diminished resistance indices and increased diastolic velocities in the middle cerebral artery (MCA) as well as a decrease in the cerebroplacental ratio, defined as the MCA resistive index divided by the resistive index of the umbilical artery [28]. Fetal anemia as a result of chronic FMH also has fetal cardiovascular consequences such as an increase in heart rate, stroke volume, cardiac output, and eventually cardiac enlargement [29].

Evaluation of FMH includes confirmation with either KB testing or flow cytometry as well as determination of fetal wellbeing. Rh immune globulin should be administered to all Rh negative women with suspected antenatal FMH in order to prevent sensitization. Immediate assessment of fetal well-being should be performed in cases of suspected FMH and the administration of steroids may be considered when delivery is anticipated prior to 34 gestational weeks. Ultrasonographic evaluation for the presence of hydrops fetalis as well as interrogation of the MCA peak systolic velocity (MCA PSV) should also be performed. MCA PSV values of greater than 1.5 MOM suggest possible fetal anemia [30]. Remote from term, cordocentesis may be offered for elevated MCA PSV values [31]. The results of antenatal testing, gestational age, and availability of trained physician personnel mitigate the decision to proceed with cordocentesis and possible intrauterine transfusion therapy. Determining the timing of subsequent transfusions in cases of FMH is challenging since the rate of ongoing FMH is variable and unpredictable. Repeat KB testing and MCA PSV measurements may be falsely low since transfused erythrocytes are partially adult in origin.

We report a unique case of FMH that demonstrated characteristics suggestive of both acute and chronic anemia. On the one hand, the deterioration of the fetal heart tracing, diminished biophysical score, decreased cord pH, and increased cord base deficit levels point to acute fetal decomposition while findings of right fetal heart enlargement, increased MCA end-diastolic velocities, reversal of flow in the aortic arch distal to the brachiocephalic vessels, and elevated immediate neonatal erythrocyte and nucleated red blood cell suggest chronic fetal compensatory mechanisms. One should consider FMH as part of the differential diagnosis for fetal or immediate neonatal anemia. It is our hope that this case will assist practitioners in differentiating acute FMH that may require emergent delivery from chronic FMH which may be able to be expectantly managed.
